# CT-derived topological intratumoral heterogeneity predicts major pathological response to neoadjuvant immunochemotherapy in resectable non-small-cell lung cancer: a two-center study

**DOI:** 10.3389/fimmu.2026.1898208

**Published:** 2026-07-27

**Authors:** Yongjie Zhou, Tingyu Hong, Hongliang Qi, Jinhong Zhao, Zhengyang Wu, Jingjing Du, Lan Liu, Fei Zou

**Affiliations:** 1 Department of Radiology, Jiangxi Cancer Hospital & Institute, Jiangxi Clinical Research Center for Cancer, The Second Affiliated Hospital of Nanchang Medical College, Nanchang, China; 2 Liaoning University of Traditional Chinese Medicine, Shenyang, China; 3 Department of Clinical Engineering, Southern Medical University, Nanfang Hospital, Guangzhou, China; 4 Department of Radiology, The Second Affiliated Hospital, Jiangxi Medical College, Nanchang University, Nanchang, China

**Keywords:** intratumoral heterogeneity, major pathological response, neoadjuvant immunochemotherapy, non-small-cell lung cancer, radiomics

## Abstract

**Objectives:**

To develop and validate a pretreatment computed tomography (CT)-derived model for predicting major pathological response (MPR) to neoadjuvant immunochemotherapy in resectable non-small-cell lung cancer (NSCLC).

**Methods:**

This multicenter retrospective study included 287 patients with stage IB-III NSCLC treated with neoadjuvant immunotherapy plus chemotherapy followed by surgery. Patients from Center A formed the training cohort (n = 224), and patients from Center B formed the external validation cohort (n = 63). Two-dimensional (2D) and three-dimensional (3D) intratumoral heterogeneity scores (ITHscores) were derived from venous-phase pretreatment CT using local radiomics, K-means clustering and connected-component topology. Candidate predictors included clinicopathological variables, ITHscores and blood-derived inflammatory indicators. Eight machine-learning algorithms were compared, and the final model was selected using cross-validation and feature ablation. Shapley additive explanations (SHAP) analysis was used to interpret feature contributions.

**Results:**

Non-MPR tumors showed significantly higher 2D and 3D ITHscores than MPR tumors in both cohorts (all P < 0.001). CatBoost was selected as the optimal algorithm. SHAP analysis identified 2D ITHscore and 3D ITHscore as the two most influential predictors, with mean absolute SHAP values of 0.403 and 0.326, respectively. The final parsimonious model retained 2D ITHscore, 3D ITHscore and systemic immune-inflammation index. It achieved AUCs of 0.824 in the training cohort and 0.792 in the external validation cohort, outperforming the clinical and ITH-only models. Calibration and decision curve analyses showed acceptable agreement and potential clinical utility.

**Conclusion:**

CT-derived topological intratumoral heterogeneity, combined with systemic inflammation, may provide a non-invasive pretreatment biomarker for predicting MPR in resectable NSCLC.

## Introduction

Neoadjuvant immunotherapy combined with chemotherapy has changed the treatment landscape for resectable non-small-cell lung cancer (NSCLC), increasing the likelihood of major pathological response (MPR) and pathological complete response (pCR) before surgery (1–3). However, treatment responses remain heterogeneous. Some tumors undergo near-complete eradication, whereas others retain substantial viable disease despite the same treatment strategy (2, 3). Because MPR and pCR can be confirmed only after resection, clinicians lack a reliable pretreatment tool for identifying patients most likely to benefit from neoadjuvant immunochemotherapy or those who may require closer monitoring, treatment adaptation or alternative strategies (4, 5).

Current pretreatment predictors address this clinical need only partially. Clinicopathological variables, programmed death-ligand 1 (PD-L1) expression, circulating inflammatory indices and conventional radiological measurements provide useful but incomplete information about tumour biology and host response (6, 7). Computed tomography (CT)-based radiomics offers a non-invasive approach for extracting quantitative imaging features from routine pretreatment scans, and has been explored for predicting MPR or pCR in patients with NSCLC receiving neoadjuvant immunochemotherapy (8–10). However, many radiomics models summarize the tumor as a single global feature vector or rely on selected handcrafted features. Such representations may overlook the spatial organization of viable tumor habitats, immune-active regions, necrosis and stromal components that collectively shape treatment sensitivity (11, 12).

Intratumoral heterogeneity is a central source of therapeutic resistance and prognostic uncertainty, and its clinical relevance may depend not only on its degree but also on its spatial topology (13). Recent imaging studies have shown that intratumoral heterogeneity scores (ITHscores), derived by integrating local radiomics, spatial clustering and connected-component topology, can non-invasively quantify tumor heterogeneity and provide biologically interpretable information across cancer types (14–16). In NSCLC, CT radiomics has also been used to predict pathological response after neoadjuvant immunochemotherapy, including habitat-based intratumoral heterogeneity models and multiregional intratumoral-peritumoral approaches (17, 18). Nevertheless, limited evidence is available on the integration of complementary 2D and 3D CT-derived topological ITHscores with blood-derived inflammatory indicators for pretreatment prediction of pathological response to neoadjuvant immunochemotherapy in NSCLC. Addressing this gap may improve pretreatment response stratification and support a more interpretable imaging-based approach for resectable NSCLC.

In this multicenter retrospective study, we developed and externally validated a pretreatment CT-based model for predicting MPR in patients with stage IB–III NSCLC receiving neoadjuvant immunotherapy plus chemotherapy. We evaluated two spatially distinct CT-derived topological ITHscores together with blood-derived inflammatory indicators and compared their performance with models based on conventional clinical variables or ITHscores alone. We investigated whether this framework could provide a non-invasive approach to pretreatment response stratification in resectable NSCLC.

## Methods

### Study design and patients

This retrospective multicenter study was approved by the Ethics Committees of Jiangxi Cancer Hospital (approval number: 2024ky217) and the Second Affiliated Hospital of Nanchang University (approval number: O-Medical Research Ethics Review (2026) No. 031), and the requirement for written informed consent was waived. Patients from Center A between June 2021 and December 2025 were used as the training cohort, whereas patients from Center B between January 2024 and June 2025 were used as an independent external validation cohort.

Eligible patients met the following criteria: (1) pathologically confirmed stage IB–III NSCLC according to the eighth edition of the American Joint Committee on Cancer (AJCC) tumor-node-metastasis (TNM) staging system; (2) receipt of at least two cycles of neoadjuvant immunotherapy combined with chemotherapy; (3) surgical resection after neoadjuvant treatment with pathological response assessment of the primary tumor and lymph nodes; and (4) contrast-enhanced chest CT performed within one month before neoadjuvant treatment. Patients were excluded if they had received previous immunotherapy, had systemic infection, autoimmune disease or hematological disease before surgery, had poor-quality images or non-measurable target lesions, or underwent biopsy before CT examination (irrespective of the interval, to avoid potential biopsy-related hemorrhagic or inflammatory changes affecting CT features). **Supplementary Figure 1** illustrates the patient enrollment process.

### CT image acquisition and tumor segmentation

Venous-phase contrast-enhanced CT images were used for all radiomics analyses because this phase was consistently available across both participating centers and provided stable tumor conspicuity for region of interest (ROI) delineation and local radiomics feature extraction. Detailed CT acquisition parameters, including scanner manufacturer, tube voltage, tube current, slice thickness and reconstruction thickness, are provided in **Supplementary Table 1**.

All pretreatment CT images were exported from the picture archiving and communication system in Digital Imaging and Communications in Medicine (DICOM) format and imported into 3D Slicer software (version 5.8.1) for tumor segmentation (19). A thoracic radiologist with 12 years of experience manually delineated the primary tumor volume while blinded to pathological response. All ROIs were reviewed by a senior thoracic radiologist with 35 years of experience. Segmentation reproducibility was assessed in 30 randomly selected patients. A second radiologist independently performed segmentation, and the original radiologist repeated the procedure after 60 days. The 2D and 3D ITHscores were recalculated using the same pipeline, and interobserver and intraobserver ICCs were calculated using a two-way random-effects, absolute-agreement, single-measure model. Tumor segmentation was performed primarily on lung-window images (width, 1500 HU; level, −600 HU), with mediastinal-window images (width, 400 HU; level, 40 HU) used as an auxiliary reference. When boundaries differed, the lung-window contour was prioritized, while the mediastinal window assisted in excluding non-tumor structures. The ROI encompassed the entire visible primary tumor, including solid, necrotic and low-attenuation components, while excluding obstructive pneumonia, atelectasis, pleural effusion, major vessels, bronchial lumen, chest wall and mediastinal structures. Cases with tumor boundaries indistinguishable from adjacent inflammation or atelectasis were excluded.

### Pathological response assessment

Postoperative pathological response was independently assessed by two pathologists experienced in thoracic tumor pathology who were blinded to radiomics results and clinical outcomes. Major pathological response (MPR) was defined as no more than 10% residual viable tumor cells in the primary tumor bed after neoadjuvant therapy. Pathological complete response (pCR) was defined as the absence of residual viable tumor cells in both the primary tumor bed and all sampled lymph nodes, namely ypT0N0 (4, 5). Discrepancies between the two pathologists were reviewed by a third senior pathologist and resolved by consensus. Interobserver agreement for MPR classification between the two initial pathologists was evaluated using Cohen’s κ coefficient, with the results provided in **Supplementary Table 2**.

### Image preprocessing

Before ITHscore calculation, CT images and masks were resampled to 1.0 × 1.0 × 1.0 mm³ using linear and nearest-neighbor interpolation, respectively. CT intensities were standardized within the tumor mask for each patient using z-score normalization, calculated as z=(x−μ)/σ, where x is the voxel intensity and μ and σ are the mean and standard deviation of CT intensities within the tumor ROI (20). The stability of intratumoral CT intensity distributions before and after z-score normalization was assessed as a preprocessing quality-control analysis, and the results are provided in **Supplementary Figure 2**. Radiomics features were extracted with a fixed bin width of 25 HU. For the 3D analysis, the whole tumor volume of interest was used. For the 2D analysis, the axial slice with the largest tumor cross-sectional area was automatically identified from the three-dimensional tumor mask and selected as the representative 2D ROI. As an acquisition-related sensitivity analysis, the 94 local radiomics features were harmonized using parametric ComBat before case-wise K-means clustering and ITHscore recalculation. Center A and Center B were specified as batches, and no MPR labels or other outcome variables were used during harmonization. All other preprocessing, window settings, K-selection procedures and model settings were unchanged.

### Construction of 2D and 3D ITHscore

**Figure 1:**
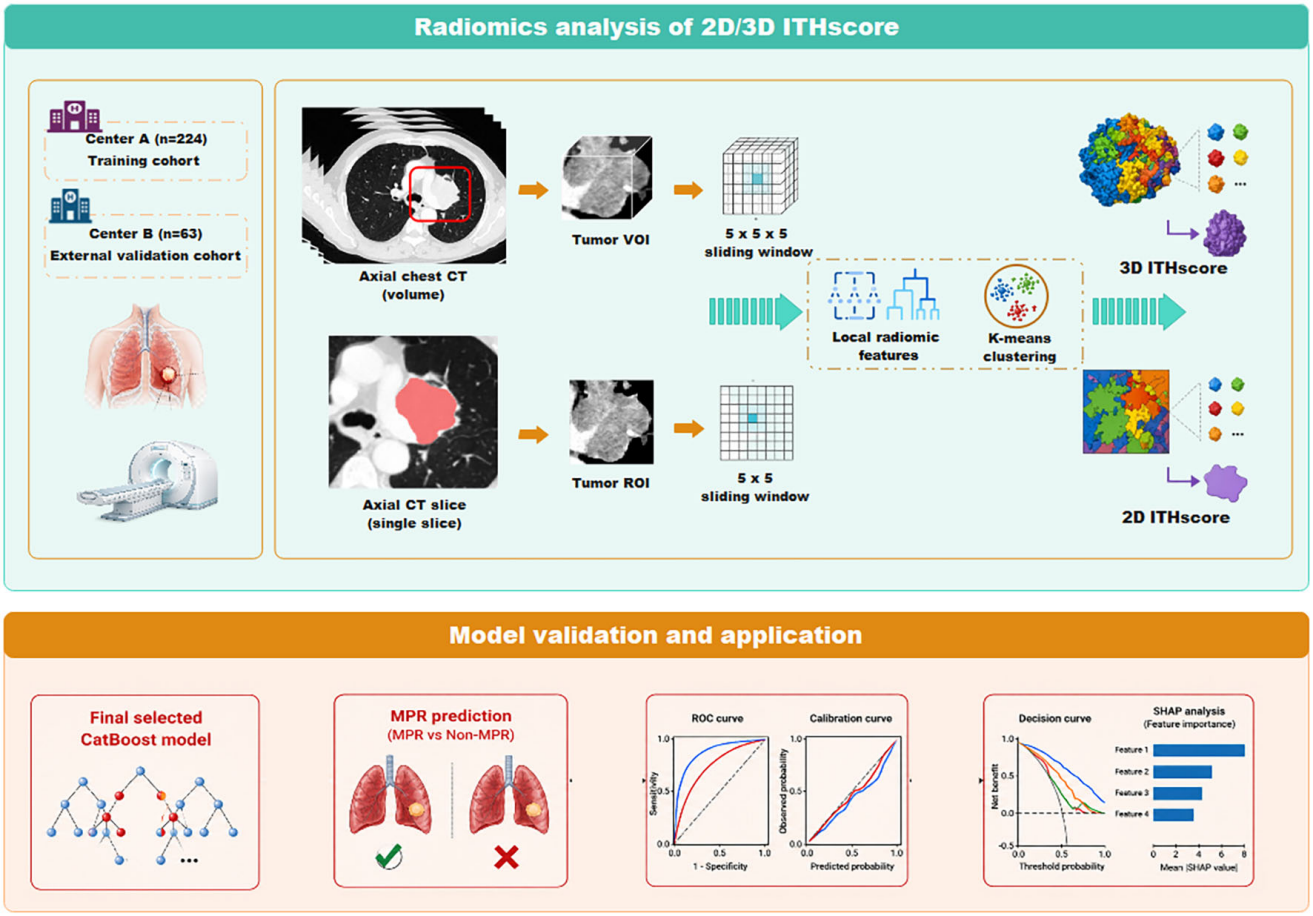
Workflow for CT-derived 2D and 3D topological intratumoral heterogeneity analysis and model development.

The 5 × 5 and 5 × 5 × 5 windows were selected to balance local spatial resolution with sufficient pixels or voxels for stable radiomics feature extraction. Sensitivity analyses were additionally performed using 3 × 3/3 × 3 × 3 and 7 × 7/7 × 7 × 7 windows. The resulting ITHscores were compared with those obtained using the primary window setting by Spearman correlation and AUC analysis. For each local subregion, 94 radiomics features were extracted using PyRadiomics, including 19 first-order features and 75 texture features (21). The ITHscore construction followed previously described topological ITHscore frameworks (22). The study workflow is shown in **Figure 1**.

For each patient, the 94-dimensional local feature vectors were standardized before K-means clustering. The optimal number of clusters was selected from K = 2–10 using the elbow method and defined as the point with the maximum perpendicular distance from the line connecting the first and last points of the within-cluster sum-of-squares curve. Sensitivity analyses using fixed K values of 4, 5 and 6 were performed to assess the robustness and comparability of the case-specific ITHscores. K-means clustering was performed with a fixed random seed, 10 initializations and a maximum of 300 iterations. The resulting cluster labels were mapped back to the tumor space to generate a 2D clustering map and a 3D clustering volume. After clustering, spatially connected components within each cluster were identified. For cluster *i*, *n_i_* denotes the number of connected components, $S_{i,max}$ denotes the area of the largest connected component in the 2D clustering map, and $V_{i,max}$ denotes the volume of the largest connected component in the 3D clustering volume. The 2D and 3D ITHscores were calculated as:


$$ITHscore=1-\frac{1}{X_{total}}\sum_{i=1}^{K}\frac{X_{i,max}}{n_{i}},\;X=\{\begin{array}{c} S,\;for\;2D\;analysis\\ V,\;for\;3D\;analysis \end{array}\}$$

A higher ITHscore indicates more spatially fragmented cluster distributions and greater intratumoral topological heterogeneity.

### Clinical variables and blood-derived indicators

Candidate predictors included general clinical variables, pathological/tumor characteristics, ITHscores and blood-derived inflammatory indicators. General clinical variables included age, sex, smoking history and drinking history. Pathological/tumor variables included tumor location, tumor size, histological type, T stage and N stage. The ITH variables included 2D ITHscore and 3D ITHscore. Blood-derived indicators included neutrophil-to-lymphocyte ratio (NLR), platelet-to-lymphocyte ratio (PLR), monocyte-to-lymphocyte ratio (MLR), lymphocyte-to-monocyte ratio (LMR), systemic immune-inflammation index (SII), systemic inflammation response index (SIRI), prognostic nutritional index (PNI) and albumin-to-globulin ratio (AGR) (7). Detailed formulas for these indicators are provided in **Supplementary Methods**.

### Model development and validation

Patients from Center A were used as the training cohort, and patients from Center B were used as the external validation cohort. The external validation cohort was not used during feature selection, hyperparameter tuning or model selection. Missing values were imputed using the mean for approximately symmetric variables and the median for skewed variables. Variable-specific missing proportions and imputation details are provided in **Supplementary Table 3**. To assess temporal robustness, patients from Center A were stratified into an early period (June 2021 to December 2023) and a late period (January 2024 to December 2025). The AUC of the final model, calculated from five-fold out-of-fold predictions, was compared between the two periods.

Eight machine-learning algorithms were compared: logistic regression (LR), support vector machine with radial basis function kernel (SVM-RBF), K-nearest neighbors (KNN), random forest (RF), ExtraTrees, AdaBoost, LightGBM and CatBoost. Continuous predictors were z-score standardized using parameters estimated within each training fold for LR, SVM-RBF and KNN, whereas tree-based algorithms, including CatBoost, were trained without standardization because they are insensitive to feature scaling. Hyperparameters were optimized using grid search with stratified five-fold cross-validation in the training cohort. The complete hyperparameter search spaces and optimal values for all eight algorithms are provided in **Supplementary Table 4**. The optimal algorithm was selected based on the highest mean five-fold cross-validation AUC in the training cohort. For the selected algorithm, features were ranked using permutation importance estimated in the training cohort and sequentially added in a top-k ablation analysis. The final parsimonious feature set was defined as the smallest subset with a cross-validation AUC within 0.01 of the maximum.

### Model evaluation and statistical analysis

Continuous variables are presented as medians with interquartile range (IQRs) and were compared using the Mann–Whitney U test. Categorical variables are reported as frequencies and percentages and were compared using the chi-square test or Fisher’s exact test, as appropriate. Model discrimination was evaluated using the AUC, accuracy, sensitivity, specificity and F1 score. The 95% confidence interval (CI) for each AUC was estimated using bootstrap resampling. AUCs were compared using the paired DeLong test. Calibration curves assessed the agreement between predicted probabilities and observed MPR status, and decision curve analysis evaluated net clinical benefit across a range of threshold probabilities. Shapley additive explanations (SHAP) were used to interpret the final selected model and quantify the contribution of each included predictor (23).

## Results

### Patient characteristics

A total of 287 patients with stage IB–III NSCLC who received neoadjuvant immunotherapy plus chemotherapy were included. The training cohort comprised 224 patients from Center A, and the external validation cohort comprised 63 patients from Center B. Baseline characteristics did not differ significantly between the cohorts (**Table 1**, all P > 0.05). Median age was 63 years (IQR, 57-68) in the training cohort and 64 years (IQR, 58-69) in the external validation cohort (P = 0.369). MPR was observed in 42.4% (95/224) and 49.2% (31/63) of patients, respectively (P = 0.337).

**Table 1:** Patient characteristics across different cohorts.

Characteristics	Center A (n=224)	Center B (n=63)	p-value
	Training cohort	External validation cohort	
Age	63 [57, 68]	64 [58, 69]	0.369
Sex			0.805
Female	20 (8.9%)	5 (7.9%)	
Male	204 (91.1%)	58 (92.1%)	
Smoking history			0.396
No	59 (26.3%)	20 (31.7%)	
Yes	165 (73.7%)	43 (68.3%)	
Drinking history			0.573
No	159 (71.0%)	47 (74.6%)	
Yes	65 (29.0%)	16 (25.4%)	
Albumin	38.81 [35.77, 41.80]	38.81 [37.72, 40.70]	0.959
Globulin	31.39 [27.70, 34.50]	31.39 [28.94, 31.39]	0.525
WBC	6.90 [6.02, 8.63]	6.76 [6.08, 7.31]	0.106
Lymphocyte	1.49 [1.18, 1.64]	1.49 [1.19, 1.70]	0.884
Monocyte	0.52 [0.41, 0.56]	0.51 [0.40, 0.57]	0.842
Platelet	285.50 [234.00, 336.50]	266.00 [212.50, 321.50]	0.433
Neutrophil	4.85 [4.28, 5.92]	4.72 [3.70, 5.20]	0.103
NLR	3.25 [2.78, 4.39]	3.25 [2.48, 4.20]	0.348
PLR	195.42 [150.87, 235.24]	174.03 [145.81, 231.21]	0.273
MLR	0.35 [0.28, 0.42]	0.35 [0.26, 0.43]	0.708
LMR	2.89 [2.40, 3.62]	2.89 [2.35, 3.78]	0.708
SII	947.79 [714.65, 1288.26]	851.14 [572.59, 1259.48]	0.105
SIRI	1.68 [1.10, 2.41]	1.62 [1.15, 2.10]	0.405
PNI	46.28 [42.98, 49.77]	46.26 [44.03, 48.20]	0.730
AGR	1.24 [1.10, 1.48]	1.24 [1.16, 1.47]	0.682
Tumor size	5.18 [4.10, 6.95]	5.10 [3.70, 6.40]	0.221
Tumor location			0.634
Left	100 (44.6%)	26 (41.3%)	
Right	124 (55.4%)	37 (58.7%)	
Histological type			0.411
Squamous cell carcinoma	180 (80.4%)	46 (73.0%)	
Adenocarcinoma	37 (16.5%)	15 (23.8%)	
Other pathological types	7 (3.1%)	2 (3.2%)	
T stage			0.462
1	11 (4.9%)	2 (3.2%)	
2	67 (29.9%)	24 (38.1%)	
3	62 (27.7%)	19 (30.2%)	
4	84 (37.5%)	18 (28.6%)	
N stage			0.076
0	25 (11.2%)	15 (23.8%)	
1	40 (17.9%)	9 (14.3%)	
2	133 (59.4%)	34 (54.0%)	
3	26 (11.6%)	5 (7.9%)	
cTNM			0.103
IB/II	28 (12.5%)	13 (20.6%)	
III	196 (87.5%)	50 (79.4%)	
Treatment response			
Non-MPR	129 (57.6%)	32 (50.8%)	0.337
MPR	95 (42.4%)	31 (49.2%)	

cTNM, clinical tumor-node-metastasis stage; WBC, white blood cell count; NLR, neutrophil-to-lymphocyte ratio; PLR, platelet-to-lymphocyte ratio; MLR, monocyte-to-lymphocyte ratio; LMR, lymphocyte-to-monocyte ratio; SII, systemic immune-inflammation index; SIRI, systemic inflammation response index; PNI, prognostic nutritional index; AGR, albumin-to-globulin ratio; MPR, major pathological response.

### Radiomics-derived ITHscore differed between MPR and non-MPR tumors

For each patient, 2D and 3D ITHscores were calculated from venous-phase CT images using local radiomics feature extraction, case-wise K-means clustering and connected-component topology analysis. Window-size sensitivity analysis demonstrated high correlations with the primary ITHscores and broadly comparable discriminatory performance across window settings in both cohorts (**Supplementary Figure 3**). The elbow-selected K values were predominantly concentrated between 4 and 6. ITHscores calculated using fixed K values showed strong correlations with the case-specific scores and broadly comparable discriminatory performance across K settings (**Supplementary Figure 4**). In the reproducibility analysis of 30 randomly selected patients, the interobserver ICCs were 0.838 (95% CI, 0.687-0.919) for the 2D ITHscore and 0.886 (95% CI, 0.775-0.944) for the 3D ITHscore. The corresponding intraobserver ICCs were 0.828 (95% CI, 0.670-0.914) and 0.898 (95% CI, 0.797-0.950), respectively, indicating good reproducibility of both ITHscore measurements (**Supplementary Table 5**). In the ComBat sensitivity analysis, the harmonized scores remained strongly correlated with the original scores in both cohorts (Spearman rho, 0.973-0.986 for 2D ITHscore and 0.985-0.993 for 3D ITHscore), and the elbow-selected K values remained concordant in 95.1% and 94.1% of cases, respectively. The association between higher ITHscores and non-MPR status was preserved in both cohorts (all P < 0.001). When the final CatBoost model was refitted using ComBat-harmonized ITHscores, the training 5-fold cross-validation AUC was 0.748 (95% CI, 0.679-0.811) and the external validation AUC was 0.794 (95% CI, 0.672-0.900), compared with 0.758 (95% CI, 0.689-0.821) and 0.792 (95% CI, 0.670-0.905) in the original analysis (**Supplementary Figure 5**).

**Figure 2:**
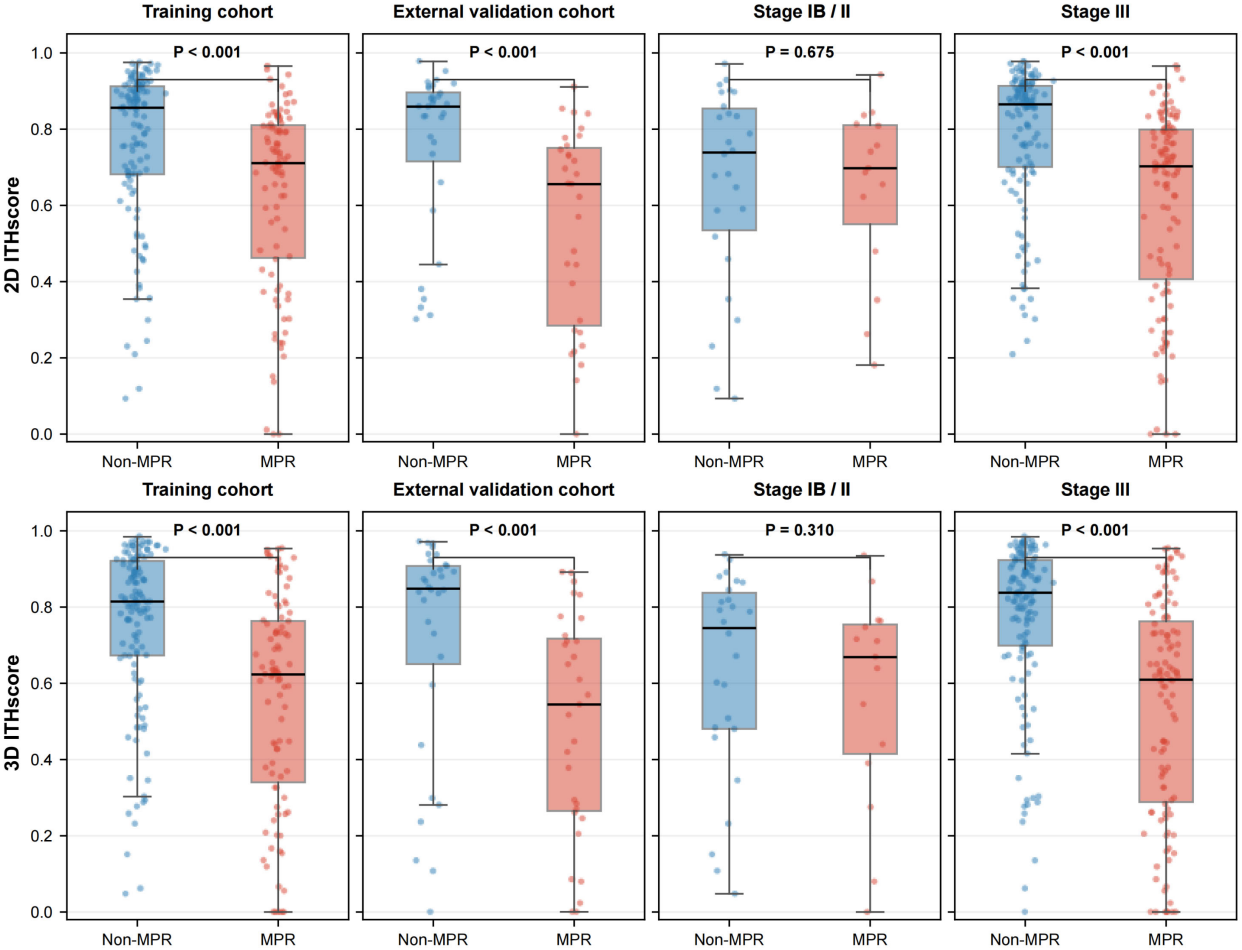
Differences in 2D and 3D ITHscores between MPR and non-MPR tumors. Boxplots show the distributions of 2D ITHscore and 3D ITHscore in non-MPR and MPR groups in the training cohort, external validation cohort, stage IB/II subgroup and stage III subgroup. Non-MPR tumors showed significantly higher 2D and 3D ITHscores than MPR tumors in both the training and external validation cohorts. This difference was also significant in patients with stage III disease, whereas no significant difference was observed in the stage IB/II subgroup. Dots represent individual patients; boxes indicate the interquartile range and median. P values were calculated using the Mann-Whitney U test.

In both cohorts, non-MPR tumors showed significantly higher ITHscores than MPR tumors (**Figure 2**). In the training cohort, the median 2D ITHscore was 0.856 (IQR, 0.682-0.912) in the non-MPR group and 0.711 (IQR, 0.462-0.810) in the MPR group (P < 0.001), while the median 3D ITHscore was 0.815 (IQR, 0.673-0.921) and 0.623 (IQR, 0.340-0.763), respectively (P < 0.001). Similar differences were observed in the external validation cohort for both 2D ITHscore (0.859 [IQR, 0.715-0.896] vs 0.656 [IQR, 0.285-0.751]; P < 0.001) and 3D ITHscore (0.848 [IQR, 0.651-0.908] vs 0.544 [IQR, 0.265-0.717]; P < 0.001). The MPR rate was 36.6% (15/41) in the stage IB/II group and 45.1% (111/246) in the stage III group (P = 0.396). When stratified by clinical stage, the discriminatory pattern was more evident in stage III disease. Among patients with stage III disease, both 2D and 3D ITHscores were significantly higher in non-MPR tumors than in MPR tumors (both P < 0.001). In contrast, neither ITHscore differed significantly between the response groups in stage IB/II disease (2D ITHscore, P = 0.675; 3D ITHscore, P = 0.310). In separate multivariable models adjusting for clinical stage and SII, both ITHscores remained independently associated with MPR (**Supplementary Table 6**).

### Comparison of candidate machine-learning algorithms

Eight machine-learning algorithms were trained and compared using the full candidate feature set (**Table 2**). CatBoost achieved the highest training five-fold cross-validation AUC (0.764; 95% CI, 0.716-0.812), with a training apparent AUC of 0.830 (95% CI, 0.773-0.881) and an external validation AUC of 0.814 (95% CI, 0.697-0.914). RF, AdaBoost and ExtraTrees showed comparable cross-validation AUCs (0.753, 0.752 and 0.743, respectively), whereas LR and KNN showed lower cross-validation performance. LightGBM yielded the highest training AUC (0.928; 95% CI, 0.889-0.959), but its external validation AUC decreased to 0.787 (95% CI, 0.660-0.895), suggesting less stable generalization. CatBoost achieved the highest mean five-fold cross-validation AUC according to the predefined algorithm-ranking criterion. The cross-validated performance of each candidate algorithm relative to the best-performing algorithm is detailed in **Supplementary Table 7**.

**Table 2:** Comparison of the efficacy of different models for predicting MPR.

Model	Training 5-fold CV					Training					External validation				
	AUC (95% CI)	Acc	Sen	Spe	F1 score	AUC (95% CI)	Acc	Sen	Spe	F1 score	AUC (95% CI)	Acc	Sen	Spe	F1 score
CatBoost	0.764 (0.716-0.812)	0.710	0.768	0.667	0.692	0.830 (0.773-0.881)	0.763	0.842	0.705	0.751	0.814 (0.697-0.914)	0.762	0.871	0.656	0.783
RF	0.753 (0.695-0.811)	0.719	0.779	0.674	0.701	0.834 (0.779-0.883)	0.768	0.811	0.736	0.748	0.785 (0.662-0.895)	0.730	0.839	0.625	0.754
AdaBoost	0.752 (0.705-0.799)	0.701	0.663	0.729	0.653	0.821 (0.765-0.874)	0.741	0.821	0.682	0.729	0.791 (0.668-0.902)	0.714	0.839	0.594	0.743
ExtraTrees	0.743 (0.684-0.801)	0.701	0.684	0.713	0.660	0.833 (0.777-0.881)	0.754	0.800	0.721	0.734	0.801 (0.678-0.912)	0.762	0.839	0.688	0.776
LightGBM	0.735 (0.674-0.796)	0.674	0.863	0.535	0.692	0.928 (0.889-0.959)	0.853	0.895	0.822	0.837	0.787 (0.660-0.895)	0.714	0.774	0.656	0.727
SVM RBF	0.721 (0.669-0.773)	0.679	0.579	0.752	0.604	0.783 (0.716-0.842)	0.768	0.579	0.907	0.679	0.768 (0.641-0.881)	0.651	0.452	0.844	0.560
LR	0.706 (0.674-0.738)	0.705	0.568	0.806	0.621	0.754 (0.685-0.816)	0.741	0.642	0.814	0.678	0.792 (0.674-0.896)	0.730	0.613	0.844	0.691
KNN	0.692 (0.644-0.739)	0.665	0.621	0.698	0.611	0.775 (0.712-0.831)	0.737	0.695	0.767	0.691	0.733 (0.607-0.851)	0.683	0.645	0.719	0.667

MPR, major pathological response; CV, cross-validation; AUC, area under the receiver operating characteristic curve; CI, confidence interval; Acc, accuracy; Sen, sensitivity; Spe, specificity; RF, random forest; SVM RBF, support vector machine with radial basis function kernel; LR, logistic regression; KNN, k-nearest neighbors; CatBoost, categorical boosting.

### Model interpretation and feature contribution

**Figure 3:**
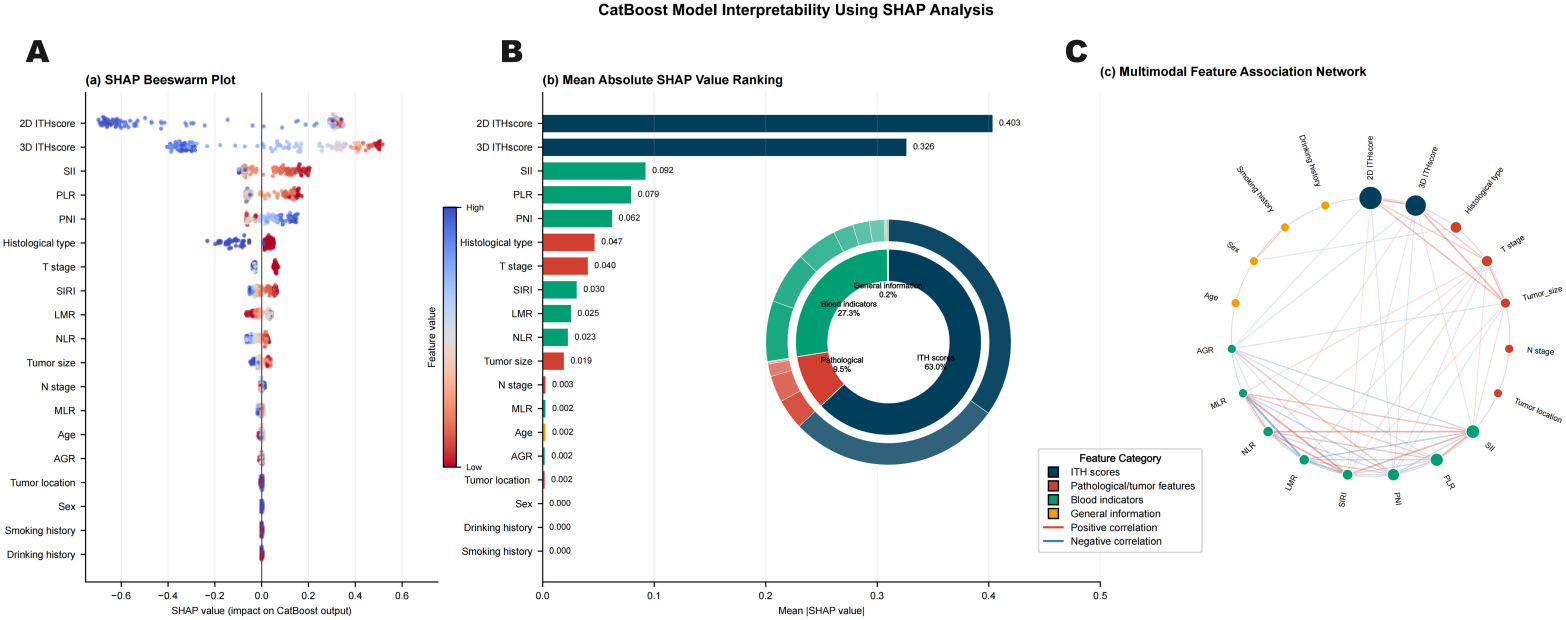
Interpretation of the CatBoost model using SHAP analysis. **(A)** SHAP beeswarm plot showing the direction and magnitude of each feature’s contribution to the CatBoost model output. Each dot represents one patient, and color indicates the feature value. **(B)** Mean absolute SHAP value ranking of candidate predictors and grouped feature-category contribution. The 2D ITHscore and 3D ITHscore were the two most influential predictors, with mean absolute SHAP values of 0.403 and 0.326, respectively. ITH-related variables accounted for 63.0% of the total feature contribution, followed by blood-derived indicators, pathological/tumor features and general clinical information. **(C)** Multimodal feature association network showing correlation structures among ITHscores, clinical tumor features and blood-derived inflammatory indicators.

SHAP analysis was performed to interpret the CatBoost model (**Figure 3**). The 2D ITHscore and 3D ITHscore were the two most influential predictors, with mean absolute SHAP values of 0.403 and 0.326, respectively. SII ranked third (mean absolute SHAP value, 0.092), followed by PLR, PNI, histological type and T stage. Feature-category analysis showed that ITH-related variables accounted for 63.0% of the total feature contribution, followed by blood indicators (27.3%), pathological or tumor-related features (9.5%) and general clinical information (0.2%). The multimodal feature association network further illustrated the correlation structure among ITHscores, tumor features and blood-derived indicators.

### Feature ablation, final model selection and validation

**Figure 4:**
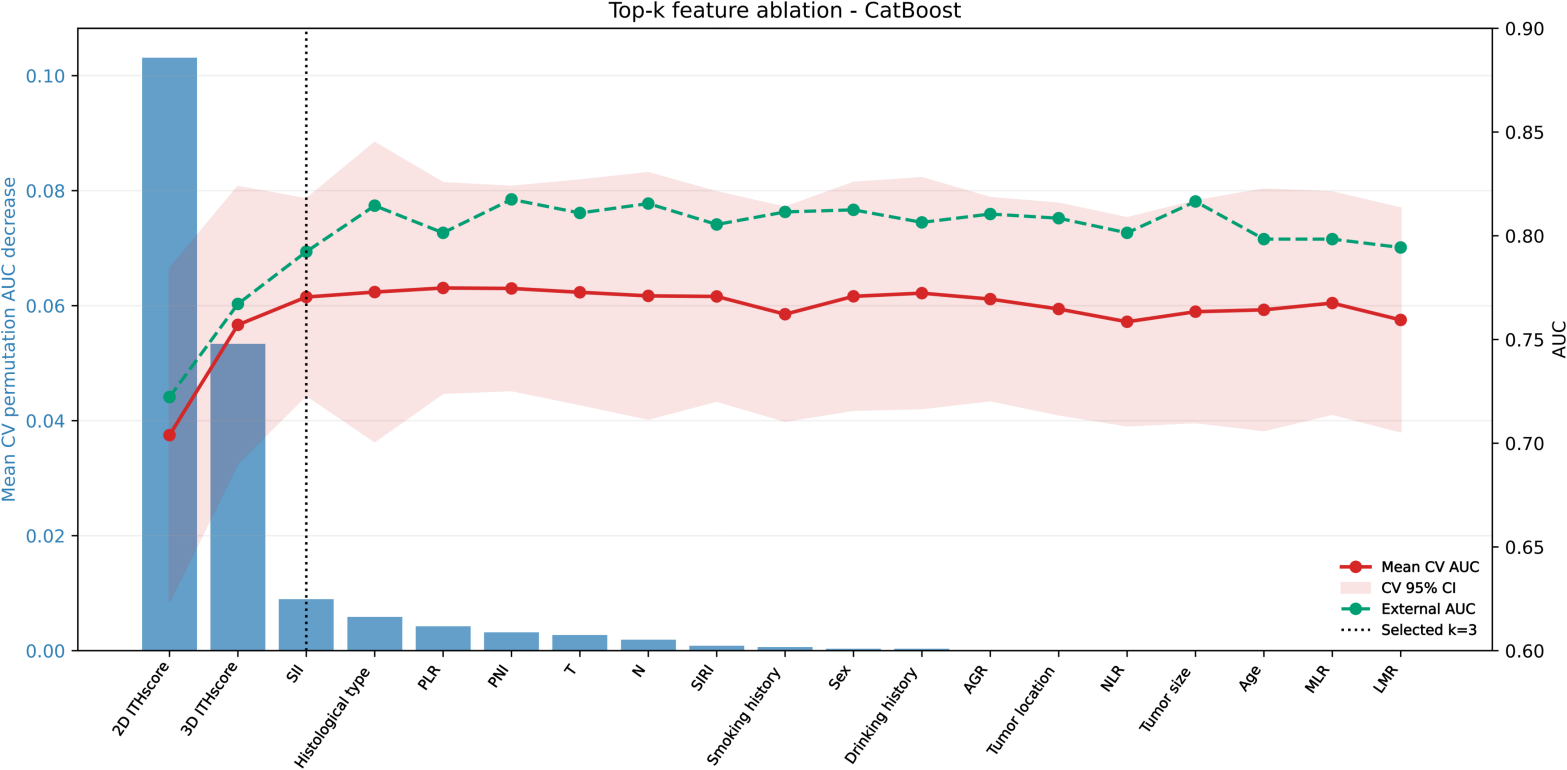
Top-k feature ablation for selecting the final CatBoost model. Features were ranked according to mean cross-validation permutation AUC decrease in the training cohort. The 2D ITHscore and 3D ITHscore produced the largest decreases in AUC when permuted, indicating their dominant contribution to model performance. The red line shows mean cross-validation AUC, the shaded area shows the 95% confidence interval, and the green dashed line shows external validation AUC as features were added sequentially. Using the predefined within-0.01 rule, the smallest feature subset close to the maximum cross-validation AUC contained three predictors: 2D ITHscore, 3D ITHscore and SII.

Top-k feature ablation was performed to identify a parsimonious feature subset for the selected CatBoost algorithm (**Figure 4**). The two ITHscore variables produced the largest decreases in cross-validation AUC when permuted, indicating their dominant contribution to model performance. The cross-validation AUC increased rapidly after adding 2D ITHscore and 3D ITHscore and then remained relatively stable after additional clinical and blood-derived variables were introduced. Using the predefined within-0.01 rule, the smallest feature subset with cross-validation performance close to the maximum consisted of three variables: 2D ITHscore, 3D ITHscore and SII. This subset was retained as the final selected combined model.

**Figure 5:**
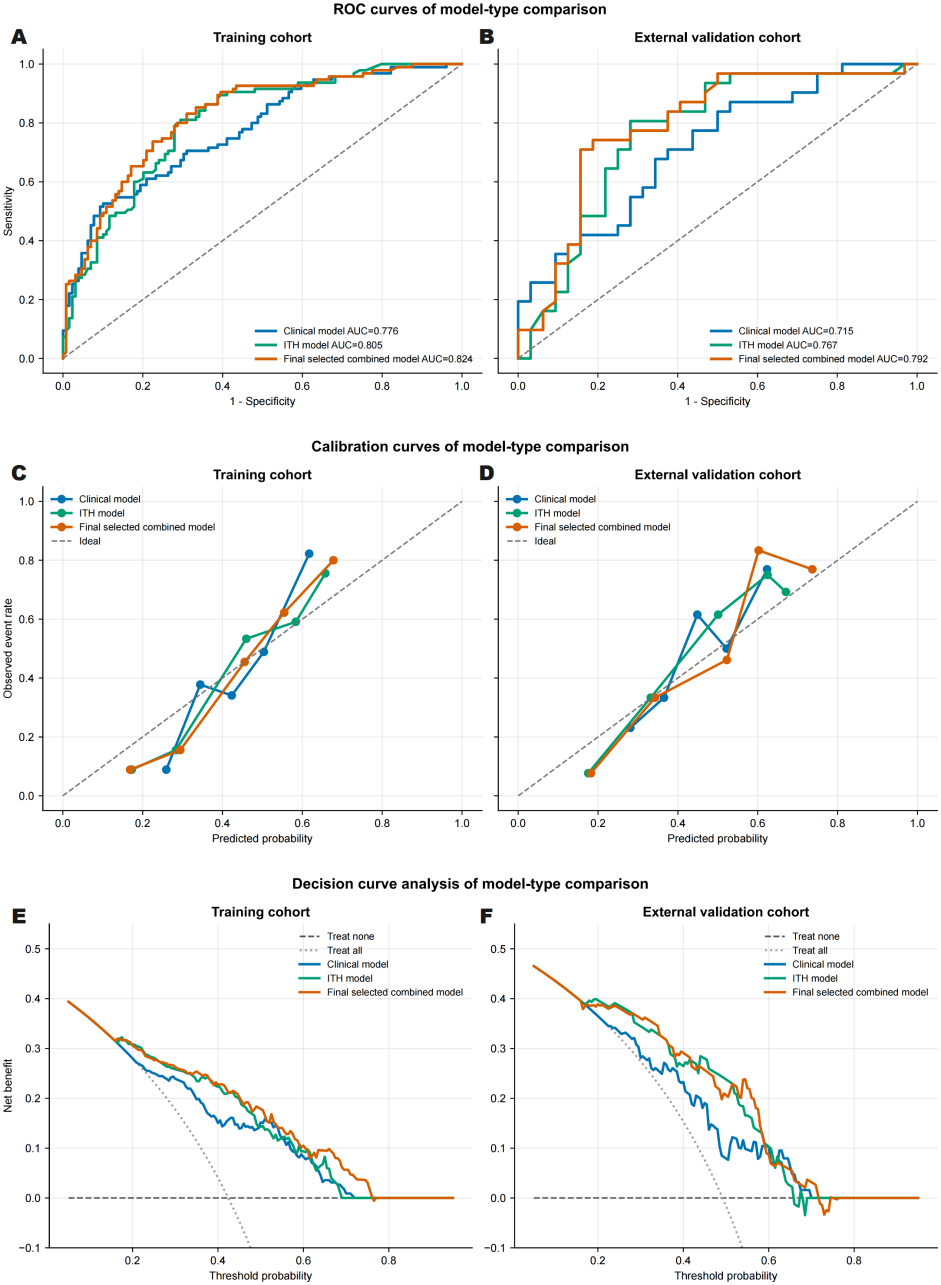
Performance comparison of the clinical, ITH and final selected combined models. **(A, B)** Receiver operating characteristic curves in the training and external validation cohorts. The final combined model achieved AUCs of 0.824 and 0.792, respectively, which were numerically higher than those of the clinical and ITH-only models. However, its improvement over the ITH-only model was not statistically significant. **(C, D)** Calibration curves showing agreement between predicted probabilities and observed MPR status. **(E, F)** Decision curve analysis showing the net benefit of each model across threshold probabilities. The final combined model showed favorable discrimination, acceptable calibration and potential clinical utility.

The clinical, ITH-only and final selected combined models were compared in the training and external validation cohorts (**Table 3**, **Figure 5**). In the training cohort, the final combined model achieved an AUC of 0.824 (95% CI, 0.766-0.877), numerically higher than those of the clinical model (AUC, 0.776; 95% CI, 0.711-0.832) and the ITH-only model (AUC, 0.805; 95% CI, 0.746-0.858). However, the difference between the final combined and ITH-only models was not statistically significant (ΔAUC, 0.019; DeLong P = 0.097; **Supplementary Table 8**). In the external validation cohort, the corresponding AUCs were 0.792 (95% CI, 0.670-0.905), 0.715 (95% CI, 0.581-0.833) and 0.767 (95% CI, 0.637-0.887), respectively. In the temporal sensitivity analysis using five-fold out-of-fold predictions from Center A, the final model showed comparable performance in the early and late periods, with AUCs of 0.751 and 0.768, respectively, and no significant prediction-by-period interaction (P = 0.986; **Supplementary Figure 6**). Calibration curves showed acceptable agreement between predicted probabilities and observed MPR status. Decision curve analysis indicated that the final combined model provided greater net benefit than the clinical model across a broad range of clinically relevant thresholds.

**Table 3:** Comparison of performance among different prediction models.

Model	Training cohort					External validation cohort				
	AUC (95% CI)	Accuracy	Sensitivity	Specificity	F1 score	AUC (95% CI)	Accuracy	Sensitivity	Specificity	F1 score
Clinical model	0.776 (0.711-0.832)	0.741	0.526	0.899	0.633	0.715 (0.581-0.833)	0.587	0.419	0.750	0.500
ITH model	0.805 (0.746-0.858)	0.750	0.811	0.705	0.733	0.767 (0.637-0.887)	0.714	0.806	0.625	0.735
Final selected combined model	0.824 (0.766-0.877)	0.750	0.832	0.690	0.738	0.792 (0.670-0.905)	0.730	0.839	0.625	0.754

ITH, intratumoral heterogeneity; AUC, area under the receiver operating characteristic curve; CI, confidence interval.

### Representative case visualization

**Figure 6:**
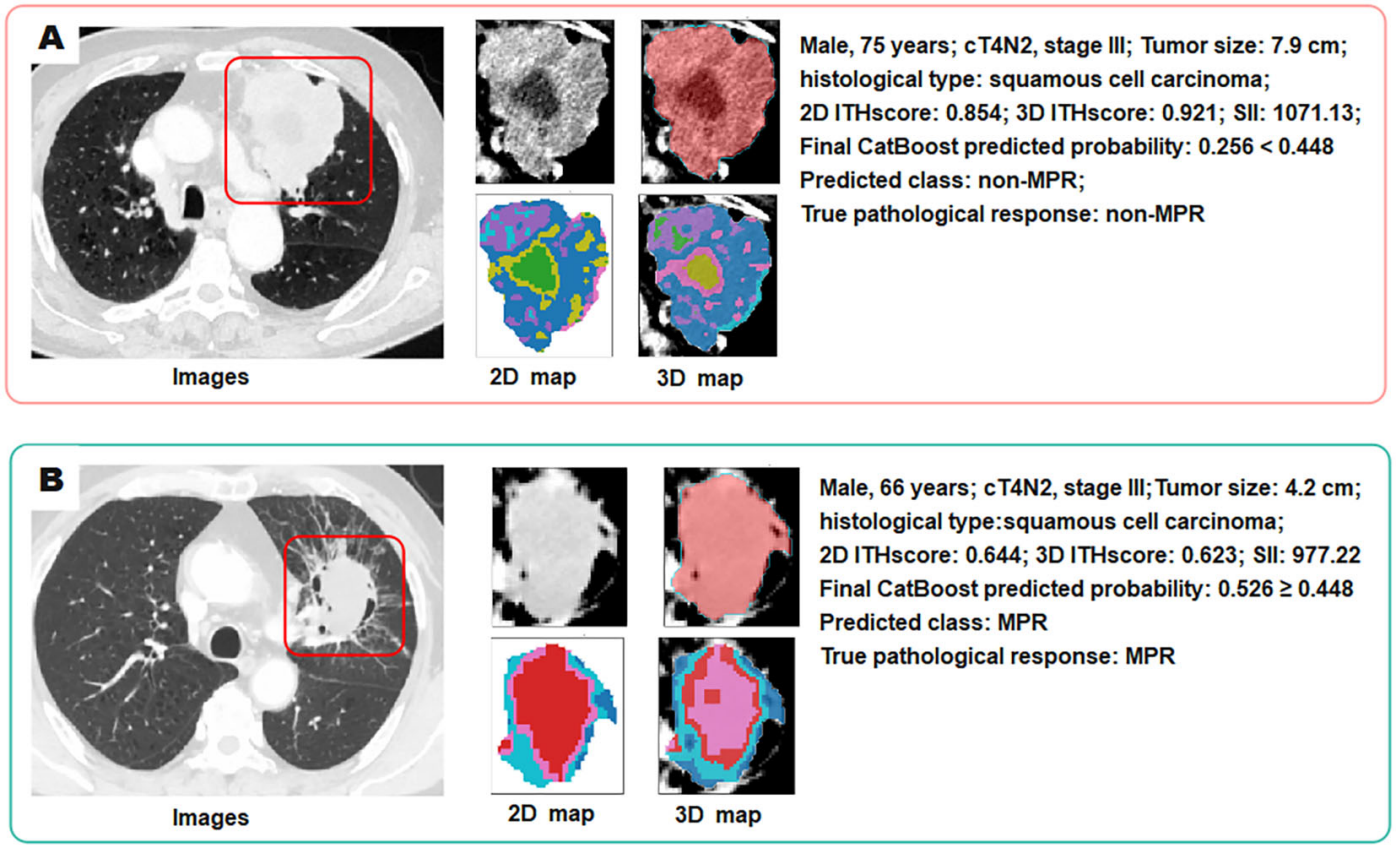
Representative cases illustrating model-based MPR prediction. **(A)** A 75-year-old male patient with stage III squamous cell carcinoma showed high topological intratumoral heterogeneity, with a 2D ITHscore of 0.854 and a 3D ITHscore of 0.921. The final CatBoost model predicted a low probability of MPR (0.256), below the decision threshold of 0.448, and the true pathological response was non-MPR. **(B)** A 66-year-old male patient with stage III squamous cell carcinoma showed lower topological heterogeneity, with a 2D ITHscore of 0.644 and a 3D ITHscore of 0.623. The final CatBoost model predicted MPR with a probability of 0.526, above the decision threshold, consistent with the true pathological response. CT images, tumor regions of interest, 2D clustering maps and 3D clustering maps are shown for each case.

Representative cases are shown in **Figure 6**. In the non-MPR case, the tumor showed high 2D and 3D ITHscores (0.854 and 0.921, respectively), and the final CatBoost model assigned a low predicted probability of MPR (0.256), below the training-derived threshold of 0.448. In contrast, the MPR case showed lower 2D and 3D ITHscores (0.644 and 0.623, respectively), and the final model assigned a higher predicted probability of MPR (0.526), above the decision threshold. These examples visually illustrate how lower topological heterogeneity patterns were associated with MPR prediction in the final model.

## Discussion

This multicenter study developed and externally validated a pretreatment CT-based framework for predicting MPR after neoadjuvant immunotherapy plus chemotherapy in resectable NSCLC. The principal finding was that topological intratumoral heterogeneity, quantified using 2D and 3D ITHscores, was consistently higher in non-MPR tumors than in MPR tumors in both cohorts. This association was most evident in stage III disease, suggesting that topological heterogeneity may be particularly informative in anatomically advanced tumors. These findings support CT-defined topological heterogeneity as a potential pretreatment marker for identifying patients less likely to achieve MPR.

MPR and pCR remain post-resection pathological endpoints, although treatment decisions must be made before surgery. In a real-world multicenter cohort of 158 patients with stage IB-IIIB NSCLC receiving neoadjuvant chemoimmunotherapy, MPR and pCR were achieved in 60.1% and 39.2% of patients, respectively, and both endpoints were significantly associated with improved progression-free and overall survival (24). In another cohort of 368 patients with NSCLC treated with neoadjuvant chemotherapy, MPR occurred in only 12.0% of patients but was associated with better overall survival (HR, 0.36; 95% CI, 0.17-0.77) and disease-free survival (HR, 0.59; 95% CI, 0.36-0.92) (25). These findings underscore the clinical need for non-invasive pretreatment markers that can help identify patients who are more or less likely to achieve meaningful pathological regression.

Building on previous NSCLC imaging heterogeneity studies, the present study extends CT-derived topological heterogeneity analysis to pretreatment response stratification after neoadjuvant immunochemotherapy. Li et al. originally proposed an ITHscore in NSCLC by integrating local radiomic features with global pixel distribution patterns, and showed that this metric was associated with tumour invasiveness, pathological phenotypes and survival across multiple cohorts (26). More recent studies have applied CT-based heterogeneity analysis to the neoadjuvant immunochemotherapy setting. Ye et al. reported that an intratumoral heterogeneity habitat model outperformed a traditional radiomics model for pCR prediction, with higher AUCs in both the training cohort and external validation cohort (18). Multiregional intratumoral-peritumoral radiomics studies have also suggested that spatial information around and within the tumor may improve pathological response prediction in NSCLC (9, 17). Unlike these studies, our work specifically evaluated whether complementary 2D and 3D topological ITHscores, combined with systemic inflammatory information, could provide clinically useful pretreatment stratification before postoperative pathological response is available.

The 2D and 3D ITHscores provided spatially distinct but related descriptions of tumor heterogeneity. The largest-slice 2D ITHscore captured dominant cross-sectional heterogeneity, whereas the 3D ITHscore quantified volumetric fragmentation across the entire lesion, consistent with previous topological ITH studies (14, 15). Greater fragmentation of CT-defined subregions may reflect regional differences in tumor cellularity, necrosis, stromal remodeling, vascular perfusion and immune-cell infiltration (11–13). Such microenvironmental diversity may create tumor subregions with differing sensitivities to immunochemotherapy, although these mechanisms were not directly evaluated here. The two ITHscores were the most influential predictors in the SHAP analysis and accounted for 63.0% of the total feature contribution. Feature ablation also showed that the principal gain in model performance occurred after adding these two variables. Thus, the paired ITHscores carried most of the response-related predictive information; their interpretation does not depend on a significant incremental benefit from SII.

The final model retained SII together with the two ITHscores, yielding a compact three-feature model. Among the blood-derived indicators, SII showed the strongest contribution and was the only inflammatory marker retained after feature ablation. Other indices, including PLR, PNI, SIRI, NLR and LMR, also appeared in the feature-importance ranking, suggesting that systemic inflammatory and nutritional status may be related to pathological response (27–29). Systemic inflammatory markers reflect the balance among neutrophil- and platelet-related tumor-promoting activity, lymphocyte-mediated antitumor immunity and nutritional status. These processes may influence angiogenesis, immune evasion and treatment sensitivity, providing a plausible link between circulating inflammatory indices and imaging-defined tumor phenotypes. Recent studies in hepatocellular carcinoma and oesophageal squamous cell carcinoma similarly showed that integrating serum inflammatory-immune biomarkers with CT radiomics improved characterization of microvascular invasion and pathological grade, respectively (30, 31). Although these studies involved other tumor types, they support a broader relationship between host systemic inflammation and radiologically expressed tumor behavior. Nevertheless, SII provided only modest incremental discrimination beyond the ITHscores, and the improvement was not statistically significant. The ITH-only model achieved AUCs of 0.805 in the training cohort and 0.767 in the external validation cohort, only modestly lower than those of the final combined model. These findings indicate that the ITHscores themselves may already capture most of the imaging-accessible host-tumor interaction information associated with treatment response. The parsimonious ITH-only model may therefore be useful when laboratory information is unavailable, although its clinical utility requires confirmation in larger prospective cohorts.

Several limitations should be acknowledged. First, this retrospective study included CT images acquired using multiple scanners and reconstruction kernels. A center-level ComBat sensitivity analysis, performed without outcome labels on the local radiomics features before ITHscore recalculation, yielded highly concordant scores and similar model performance. However, scanner- and kernel-specific harmonization was not possible because case-level acquisition labels were unavailable, and residual acquisition-related variability cannot be excluded. Second, PD-L1 expression data were incomplete and could not be reliably incorporated into model development. Future prospective studies incorporating standardized PD-L1 assessment are warranted. Third, the present study did not include spatial pathology, immunohistochemistry, transcriptomics or other biological validation data. Therefore, the SHAP analysis should be interpreted as a model-level statistical explanation rather than biological or causal validation. Future studies integrating spatially matched imaging, pathological and molecular data are warranted. In addition, this study used venous-phase CT images only, and future studies should investigate whether non-contrast and arterial-phase CT provide complementary value for ITHscore-based response prediction. Finally, the relatively small external validation cohort limited the precision of performance estimates. Further validation in larger prospective multicenter cohorts is warranted.

In conclusion, pretreatment CT-derived topological intratumoral heterogeneity was associated with MPR after neoadjuvant immunochemotherapy in resectable NSCLC. The paired 2D and 3D ITHscores captured most of the response-related predictive information and retained strong performance in the external validation cohort. SII provided limited incremental discrimination. Larger prospective multicenter studies are needed to confirm the clinical value of ITHscore-based response stratification.

## Data Availability

The original contributions presented in the study are included in the article/**Supplementary Material**. Further inquiries can be directed to the corresponding authors.
