# Supplementary material for: CT-derived topological intratumoral heterogeneity predicts major pathological response to neoadjuvant immunochemotherapy in resectable non-small-cell lung cancer: a two-center study
Source: Front Immunol. 2026 Jul 27;17:1898208. doi: 10.3389/fimmu.2026.1898208 (PMC13454048; doi:10.3389/fimmu.2026.1898208)
Supplement: Supplementary file 1 [file DataSheet1.pdf]

Supplementary Material

Supplementary Methods

Patient enrolment and cohort allocation

The patient inclusion and exclusion process and allocation to the training and external validation cohorts are summarized in Figure S1.

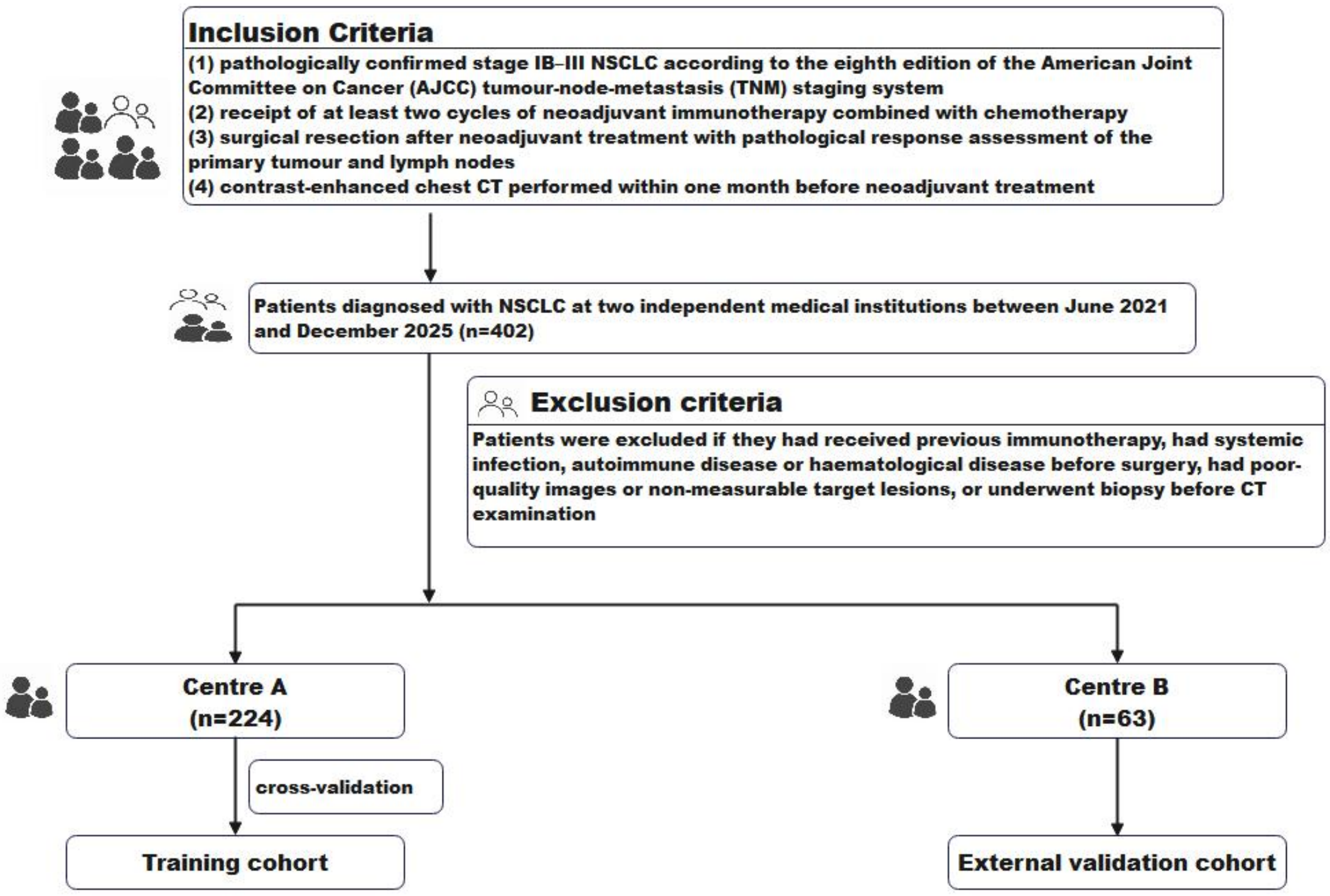

Figure S1. Flowchart of patient inclusion, exclusion, cohort allocation and model validation.

CT acquisition and preprocessing

This study used CT scanners from two independent hospitals. All patients with non-small-cell lung cancer (NSCLC) underwent pretreatment contrast-enhanced chest CT. Venous-phase images were acquired approximately 60–90 s after contrast-medium injection according to institutional protocols and were used for subsequent radiomics analysis. The scan range extended from the thoracic inlet through both adrenal glands. Detailed scanning and reconstruction parameters are provided in Table S1.

As a preprocessing quality-control analysis, voxel intensities within each tumour ROI were extracted before and after patient-wise z-score normalization. Cohort-level intensity distributions and case-level summary measures were compared between the training and external validation cohorts (Figure S2).

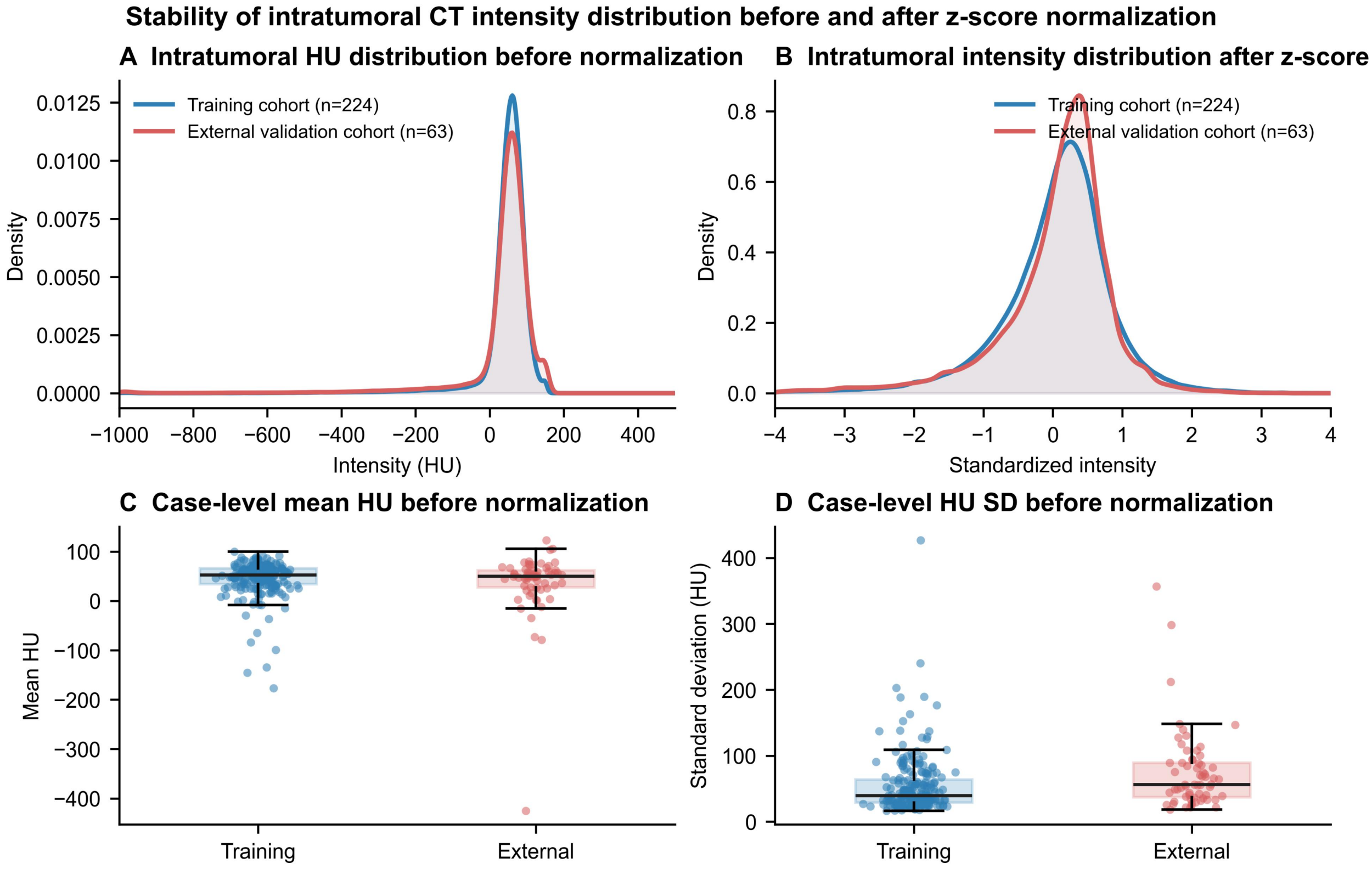

Figure S2. Intratumoral CT intensity distributions before and after patient-wise z-score normalization. (a) Pooled intratumoral HU distributions in the training and external validation cohorts before normalization. (b) Corresponding distributions after normalization. (c, d) Case-level mean and standard deviation of intratumoral HU before normalization. HU, Hounsfield unit; SD, standard deviation.

Calculation of blood-derived inflammatory and nutritional indicators

Pretreatment peripheral blood test results were collected before neoadjuvant treatment. Absolute neutrophil, lymphocyte, monocyte and platelet counts and serum albumin and globulin concentrations were used to calculate the following indicators:

Neutrophil-to-lymphocyte ratio (NLR): neutrophil count / lymphocyte count

Platelet-to-lymphocyte ratio (PLR): platelet count / lymphocyte count

Monocyte-to-lymphocyte ratio (MLR): monocyte count / lymphocyte count

Lymphocyte-to-monocyte ratio (LMR): lymphocyte count / monocyte count

Systemic immune-inflammation index (SII): platelet count × neutrophil count / lymphocyte count

Systemic inflammation response index (SIRI): neutrophil count × monocyte count / lymphocyte count

Prognostic nutritional index (PNI): serum albumin concentration + 5 × lymphocyte count

Albumin-to-globulin ratio (AGR): serum albumin concentration / serum globulin concentration

Neutrophil, lymphocyte, monocyte and platelet counts were expressed as 10^9/L; serum albumin and globulin concentrations were expressed as g/L.

Sliding-window size sensitivity analysis

To assess the influence of sliding-window size on ITHscore calculation, the complete analysis was repeated using 3 × 3 and 7 × 7 windows for the 2D analysis and 3 × 3 × 3 and 7 × 7 × 7 windows for the 3D analysis. All other preprocessing, radiomics feature-extraction, case-wise K-means clustering and connected-component topology procedures were unchanged. Alternative-window ITHscores were compared with the primary 5 × 5 and 5 × 5 × 5 ITHscores using Spearman correlation and receiver operating characteristic analysis.

All 287 cases were successfully processed for each window setting. Compared with the primary setting, Spearman correlation coefficients were 0.803 and 0.865 for the smaller 2D and 3D windows, respectively, and 0.788 and 0.852 for the larger windows, respectively (all $P < 0.001$). Discrimination remained broadly comparable across window sizes in both cohorts (Figure S3).

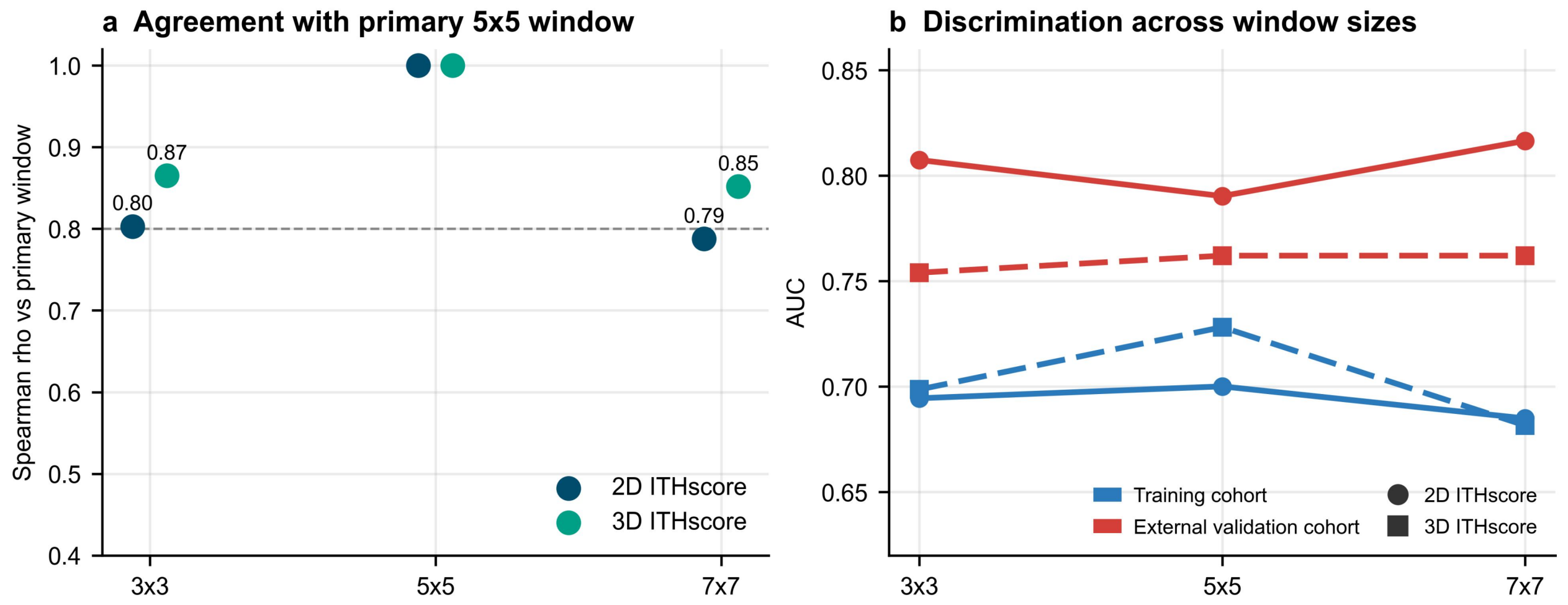

Figure S3. Sensitivity analysis of sliding-window size for 2D and 3D ITHscore calculation. (a) Spearman correlations between ITHscores obtained using alternative windows and those obtained using the primary 5 × 5 and 5 × 5 × 5 settings. (b) AUCs of 2D and 3D ITHscores across window sizes in the training and external validation cohorts. Circles with solid lines denote 2D ITHscore; squares with dashed lines denote 3D ITHscore. AUC, area under the receiver operating characteristic curve.

K-value selection and sensitivity analysis

For each patient, candidate K values from 2 to 10 were evaluated using the K-means within-cluster sum of squares. The elbow was defined as the point with the maximum perpendicular distance from the straight line joining the first and last points of the inertia curve. To assess comparability across case-specific K values, 2D and 3D ITHscores were recalculated using fixed K values of 4, 5 and 6, which covered 99.3% and 99.7% of the elbow-selected values in the 2D and 3D analyses, respectively.

Fixed-K scores showed strong correlations with the case-specific elbow-selected scores (Spearman ρ, 0.886–0.942 for 2D ITHscore and 0.917–0.969 for 3D ITHscore; all $P < 0.001$), with broadly comparable discriminatory performance across K settings in both cohorts (Figure S4).

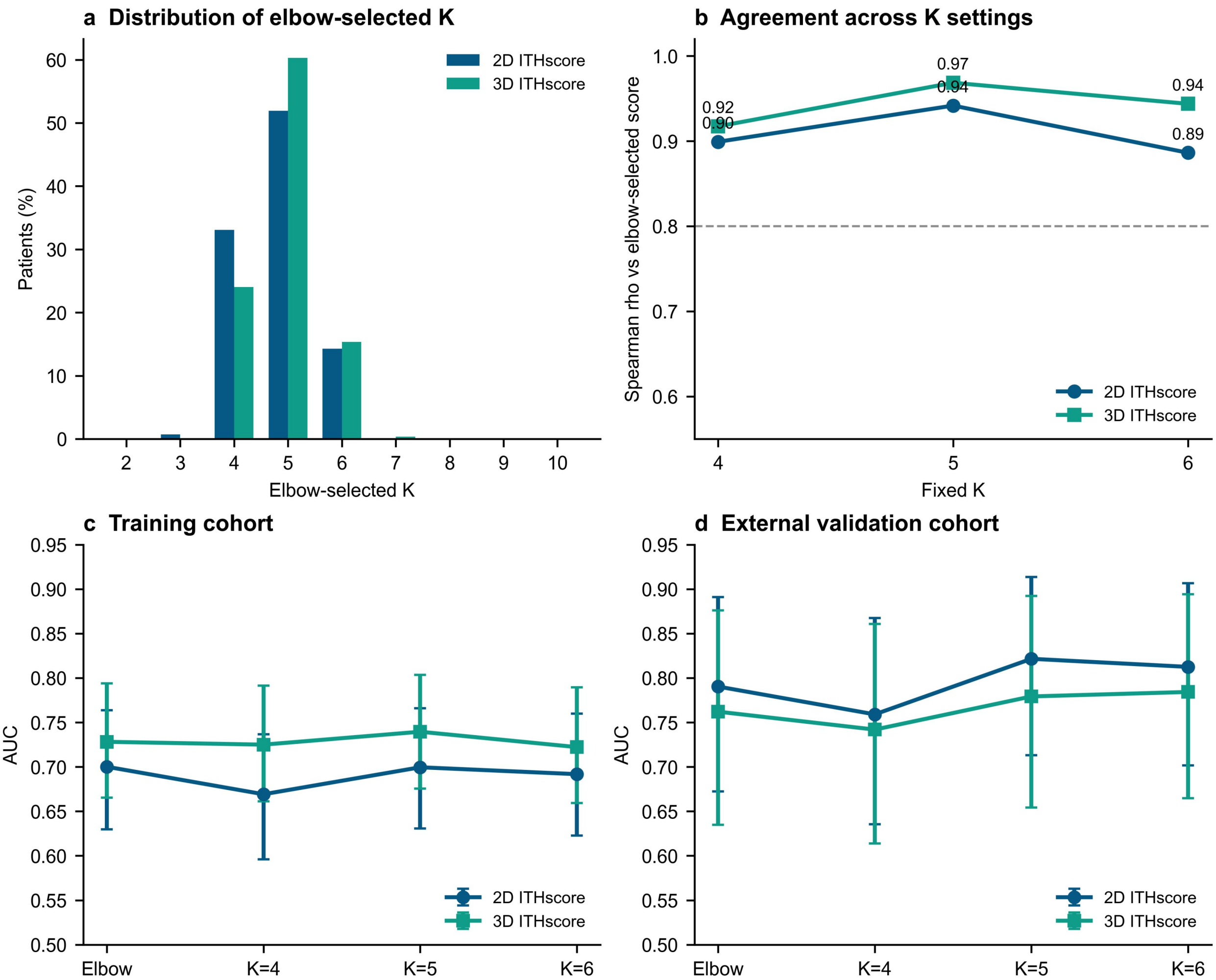

Figure S4. Distribution and sensitivity analysis of K values used for ITHscore calculation. (a) Distribution of case-specific elbow-selected K values for 2D and 3D analyses. (b) Spearman correlations between fixed-K and elbow-selected ITHscores. (c, d) AUCs and 95% confidence intervals for elbow-selected and fixed-K ITHscores in the training and external validation cohorts, respectively. AUC, area under the receiver operating characteristic curve.

ComBat harmonization sensitivity analysis

To evaluate the robustness of ITHscore calculation to center-level acquisition-related variation, parametric ComBat harmonization was applied to the 94 local radiomics features before case-wise K-means clustering. Center A and Center B were specified as batches. Harmonization was performed without using MPR labels or other outcome variables, and all remaining ITHscore and modelling settings were unchanged. The harmonized 2D and 3D ITHscores remained strongly correlated with the original scores in both cohorts (Spearman rho, 0.973-0.986 for 2D ITHscore and 0.985-0.993 for 3D ITHscore). The corresponding K values remained concordant in 95.1% and 94.1% of cases. The final model retained comparable training and external validation performance after harmonization (Figure S5).

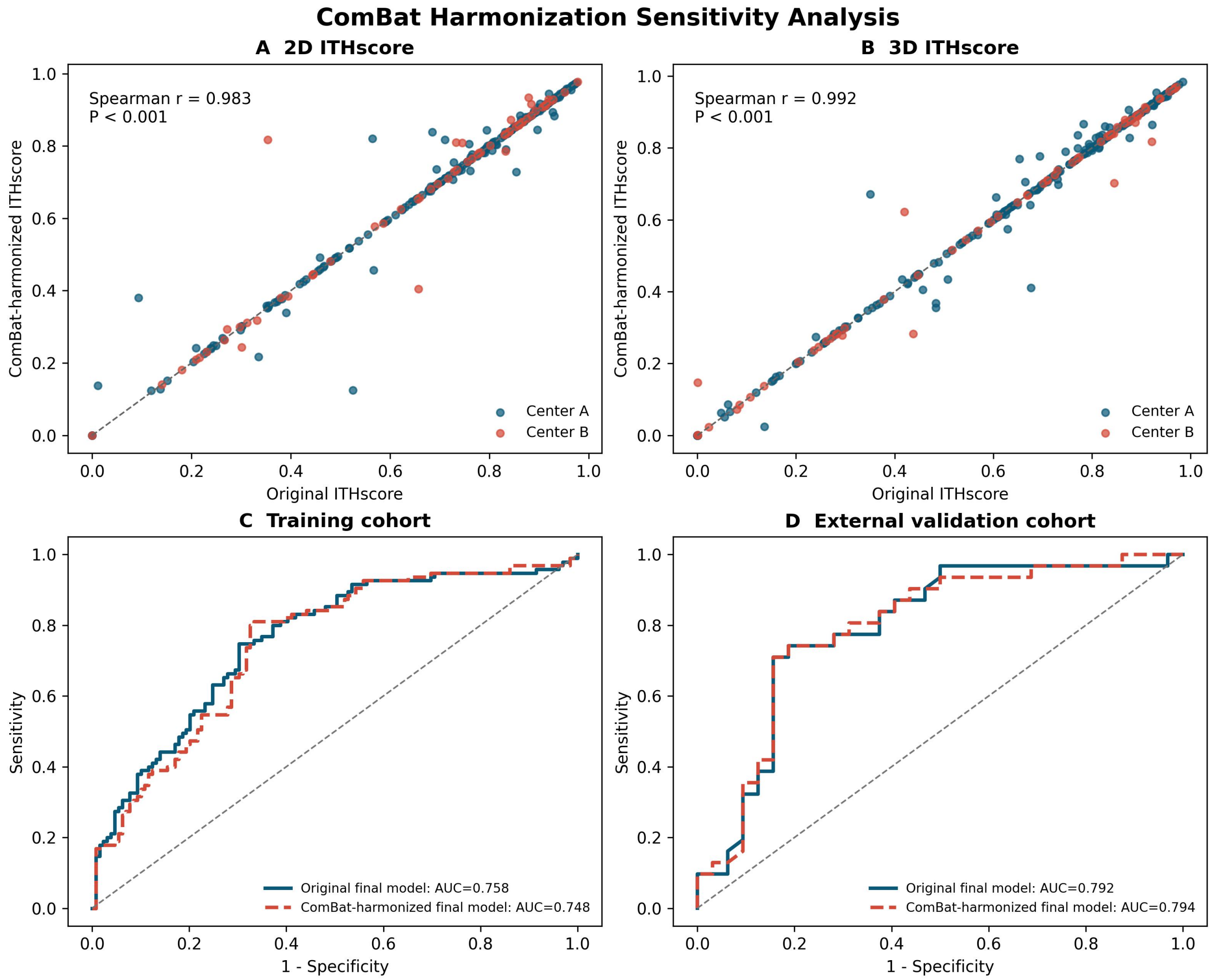

Figure S5. ComBat harmonization sensitivity analysis. (a, b) Correlations between the original and ComBat-harmonized 2D and 3D ITHscores. Points are coloured by center. (c, d) Receiver operating characteristic curves of the original and ComBat-harmonized final CatBoost models in the training cohort and external validation cohort. ComBat was applied to local radiomics features before case-wise clustering without using outcome labels. AUC, area under the receiver operating characteristic curve.

Temporal sensitivity analysis

To assess temporal robustness within Center A, patients were divided into an early period (June 2021 to December 2023; n = 89) and a late period (January 2024 to December 2025; n = 135). MPR rates and the discrimination of the 2D ITHscore, 3D ITHscore and final model were compared between periods using five-fold out-of-fold predictions. A prediction-by-period interaction term was tested in logistic regression.

MPR rates were 42.7% and 42.2% in the early and late periods, respectively (P = 1.000). The final model achieved AUCs of 0.751 and 0.768, respectively; no significant prediction-by-period interaction was observed (P = 0.986; Figure S6).

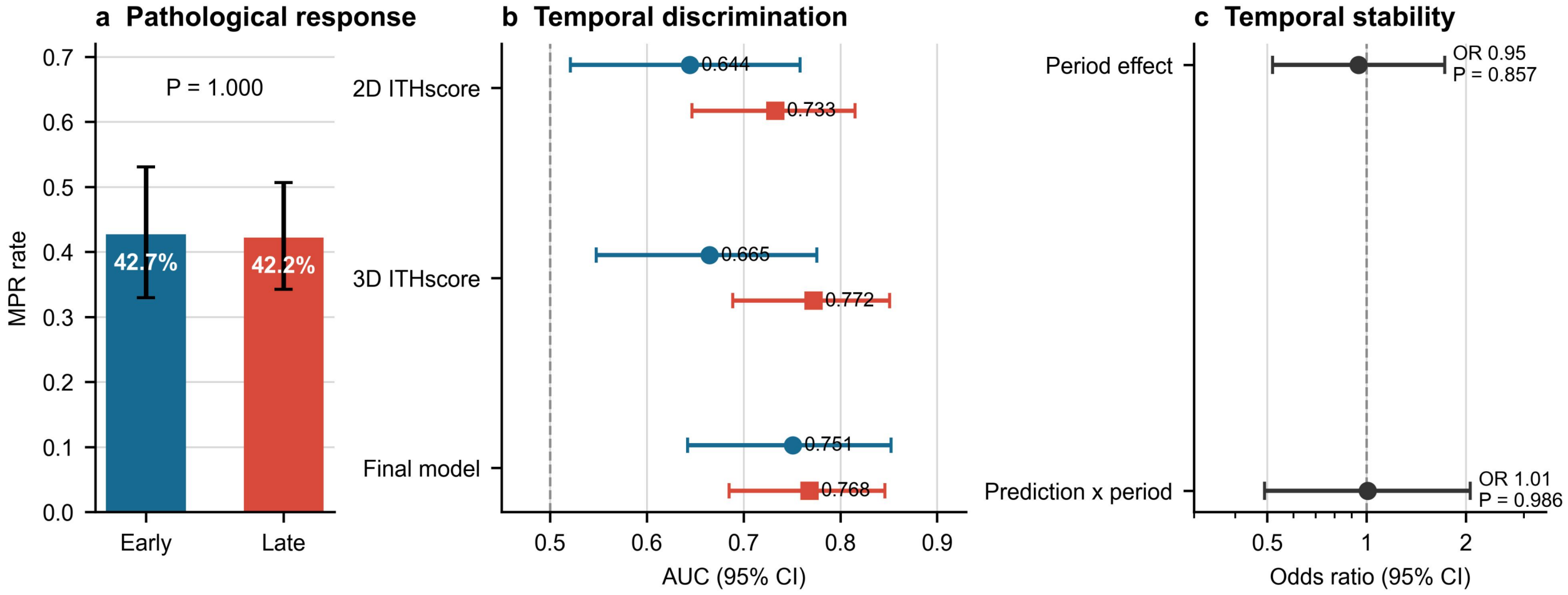

Figure S6. Temporal sensitivity analysis within Center A. (a) MPR rates in the early and late study periods. (b) AUCs and 95% confidence intervals for the 2D ITHscore, 3D ITHscore and final model in each period. Blue circles and red squares indicate the early and late periods, respectively. (c) Odds ratios and 95% confidence intervals for the period effect and the prediction-by-period interaction. AUC, area under the receiver operating characteristic curve; CI, confidence interval; MPR, major pathological response; OR, odds ratio.

Supplementary Tables

Table S1. CT scanning protocol for patients with NSCLC.

Parameter	SIEMENS	SIEMENS	GE	SIEMENS
CT scanner	CT64	CT64	CT64	CT128
Scanner model	SOMATOM Definition Edge	SOMATOM Definition AS	Revolution Frontier	SOMATOM Definition Edge
Scan phase	Contrast-enhanced venous phase	Contrast-enhanced venous phase	Contrast-enhanced venous phase	Contrast-enhanced venous phase
Tube voltage (kV)	120	120	120	120
Tube current (mA)	200	200	300	200
Rotation time (s)	0.500	0.500	0.500	0.500
Collimation (mm)	64 × 0.6	64 × 0.6	64 × 0.625	64 × 0.6
Slice thickness (mm)	1	1	1.250	1
Matrix	512 × 512	512 × 512	512 × 512	512 × 512
Field of view (mm)	350	350	350	350
Reconstruction kernel	B30f	B30f	Standard	B30f

CT, computed tomography; NSCLC, non-small-cell lung cancer.

Table S2. Interobserver agreement for MPR assessment between the two pathologists.	
**Agreement metric**	**Value**
Overall agreement	0.958
Cohen's kappa	0.915
95% CI	0.868-0.962
P value	<0.001
CI, confidence interval; MPR, major pathological response.	

Table S3. Missingness and imputation of source laboratory variables.				
**Variable**	**Missing, n**	**Missing, %**	**Imputation method**	**Imputed value**
Albumin	46	16.0	Mean	38.813
Globulin	53	18.5	Mean	31.386
WBC	35	12.2	Median	6.900
Lymphocyte	51	17.8	Mean	1.493
Monocyte	53	18.5	Mean	0.516
Platelet	29	10.1	Mean	291.756
Neutrophil	52	18.1	Median	4.850

WBC, white blood cell count.

Table S4. Hyperparameter optimization for the eight candidate machine-learning algorithms.

Algorithm	Fixed settings	Hyperparameter search space	Optimal values
LR	solver=liblinear; max_iter=5000; class_weight=balanced; random_state=77	C: 0.01, 0.1, 1.0, 10.0	C=0.01
SVM-RBF	kernel=rbf; probability=True; class_weight=balanced; random_state=77	C: 0.1, 1.0, 10.0; gamma: scale, 0.01, 0.1	C=0.1; gamma=scale
KNN	Minkowski distance	n_neighbors: 3, 5, 7, 9, 11, 15; weights: uniform, distance; p: 1, 2	n_neighbors=15; weights=uniform; p=1
RF	n_estimators=300; class_weight=balanced; random_state=77; n_jobs=1	max_depth: 2, 3, None; min_samples_leaf: 2, 5	max_depth=2; min_samples_leaf=5
ExtraTrees	random_state=77; n_jobs=1	n_estimators: 500, 800; criterion: gini, entropy; max_depth: 2, 3, 4; min_samples_leaf: 2, 5, 10; min_samples_split: 2, 10; max_features: sqrt, 0.5, None; bootstrap: False; class_weight: balanced	n_estimators=800; criterion=entropy; max_depth=3; min_samples_leaf=10; min_samples_split=2; max_features=None; bootstrap=False; class_weight=balanced
AdaBoost	random_state=77	n_estimators: 50, 100; learning_rate: 0.05, 0.1, 0.5	n_estimators=100; learning_rate=0.05
LightGBM	objective=binary; class_weight=balanced; random_state=77; n_jobs=1	n_estimators: 80, 150; num_leaves: 7, 15; learning_rate: 0.03, 0.1	n_estimators=80; num_leaves=7; learning_rate=0.03
CatBoost	loss_function=Logloss; eval_metric=AUC; random_seed=77; verbose=False; allow_writing_files=False	iterations: 100, 200; depth: 2, 3; learning_rate: 0.03, 0.1	iterations=100; depth=2; learning_rate=0.03

CV, cross-validation; KNN, K-nearest neighbours; LR, logistic regression; RF, random forest; RBF, radial basis function; SVM, support vector machine.

Table S5. Interobserver and intraobserver reproducibility of 2D and 3D ITHscores.			
**Analysis**	**Metric**	**ICC**	**95% CI**
Interobserver	2D ITHscore	0.838	0.687-0.919
Interobserver	3D ITHscore	0.886	0.775-0.944
Intraobserver	2D ITHscore	0.828	0.670-0.914
Intraobserver	3D ITHscore	0.898	0.797-0.950

ICC, intraclass correlation coefficient; CI, confidence interval; ITHscore, intratumoral heterogeneity score. ICCs were calculated using a two-way random-effects, absolute-agreement, single-measure model in 30 randomly selected patients.

Table S6. Stage-specific MPR rates and stage-adjusted multivariable analyses.

A. Stage-specific MPR rates

Cohort	Stage IB/II, n	Stage IB/II MPR, n (%)	Stage III, n	Stage III MPR, n (%)	P value
Overall cohort	41	15 (36.6%)	246	111 (45.1%)	0.396
Training cohort	28	10 (35.7%)	196	85 (43.4%)	0.541
External validation cohort	13	5 (38.5%)	50	26 (52.0%)	0.536

B. Multivariable logistic regression for MPR in the training cohort

Model	Predictor	OR	95% CI lower	95% CI upper	P value
2D ITHscore model	2D ITHscore (per SD)	0.534	0.393	0.726	<0.001
2D ITHscore model	SII (per SD)	0.435	0.228	0.829	0.011
2D ITHscore model	Stage III vs stage IB/II	1.968	0.807	4.798	0.136
3D ITHscore model	3D ITHscore (per SD)	0.428	0.308	0.596	<0.001
3D ITHscore model	SII (per SD)	0.459	0.236	0.891	0.021
3D ITHscore model	Stage III vs stage IB/II	2.280	0.904	5.751	0.081

CI, confidence interval; ITH, intratumoral heterogeneity; MPR, major pathological response; OR, odds ratio; SD, standard deviation; SII, systemic immune-inflammation index.

Table S7. Cross-validated performance of candidate algorithms relative to the best-performing algorithm.			
**Algorithm**	**Mean 5-fold CV AUC (95% CI)**	**AUC difference from best**	**Within 0.01 of best**
CatBoost	0.764 (0.716, 0.812)	——	Yes
RF	0.753 (0.695, 0.811)	0.0110	No
AdaBoost	0.752 (0.705, 0.799)	0.0115	No
ExtraTrees	0.743 (0.684, 0.801)	0.0211	No
LightGBM	0.735 (0.674, 0.796)	0.0290	No
SVM-RBF	0.721 (0.669, 0.773)	0.0428	No
LR	0.706 (0.674, 0.738)	0.0582	No
KNN	0.692 (0.644, 0.739)	0.0721	No

AUC, area under the receiver operating characteristic curve; CI, confidence interval; CV, cross-validation; KNN, K-nearest neighbours; LR, logistic regression; RF, random forest; SVM-RBF, support vector machine with radial basis function kernel. Classification relative to the 0.01 threshold was based on unrounded AUC values.

**Table S8. Formal comparison of the final combined model and ITH-only model.**				
**Dataset**	**Final combined model AUC**	**ITH-only model AUC**	**ΔAUC (95%CI)**	**DeLong P value**
Training 5-fold CV (OOF)	0.758	0.742	0.016 (−0.009, 0.041)	0.202
Training apparent	0.824	0.805	0.019 (−0.003, 0.042)	0.097
External validation	0.792	0.767	0.025 (−0.017, 0.067)	0.240
AUC, area under the receiver operating characteristic curve; CI, confidence interval; CV, cross-validation; ITH, intratumoral heterogeneity; OOF, out-of-fold.				
